# PNC-27, a Chimeric p53-Penetratin Peptide Binds to HDM-2 in a p53 Peptide-like Structure, Induces Selective Membrane-Pore Formation and Leads to Cancer Cell Lysis

**DOI:** 10.3390/biomedicines10050945

**Published:** 2022-04-20

**Authors:** Ehsan Sarafraz-Yazdi, Stephen Mumin, Diana Cheung, Daniel Fridman, Brian Lin, Lawrence Wong, Ramon Rosal, Rebecca Rudolph, Matthew Frenkel, Anusha Thadi, William F. Morano, Wilbur B. Bowne, Matthew R. Pincus, Josef Michl

**Affiliations:** 1 NomoCan Pharmaceuticals LLC, New York Blood Center, 310 East 67th Street, New York, NY 10065, USA; stephen.mumin@nomocan.com; 2 Department of Pathology, SUNY Downstate Medical Center, 450 Clarkson Avenue, Brooklyn, NY 11203, USA; diana.cheung@downstate.edu (D.C.); daniel_fridman@g.harvard.edu (D.F.); bl538@cornell.edu (B.L.); lcwong@mit.edu (L.W.); jmichl@downstate.edu (J.M.); 3 Department of Health of New York City, 455 First Avenune, New York, NY 10016, USA; rvr123@gmail.com; 4 Microscopy and Imaging Department, American Museum of Natural History, Central Park West and 79th Street, New York, NY 10024, USA; rebecca.rudolph@ge.com (R.R.); matthew.frenkel@nyu.edu (M.F.); 5 Department of Surgery, Drexel University College of Medicine, 230 North Broad Street, Philadelphia, PA 19102, USA; anusha.thadi@gmail.com (A.T.); wmorano@llu.edu (W.F.M.); 6 Department of Surgery, Sidney Kimmel Medical College, Thomas Jefferson University, 1100 Walnut Street, Philadelphia, PA 19107, USA

**Keywords:** PNC-27, p53 binding domain, cell-penetrating peptides (CPP), HDM-2/MDM-2, plasma membrane pore formation

## Abstract

PNC-27, a 32-residue peptide that contains an HDM-2 binding domain and a cell-penetrating peptide (CPP) leader sequence kills cancer, but not normal, cells by binding to HDM-2 associated with the plasma membrane and induces the formation of pores causing tumor cell lysis and necrosis. Conformational energy calculations on the structure of PNC-27 bound to HDM-2 suggest that 1:1 complexes form between PNC-27 and HDM-2 with the leader sequence pointing away from the complex. Immuno-scanning electron microscopy was carried out with cancer cells treated with PNC-27 and decorated with an anti-PNC-27 antibody coupled to 6 nm gold particles and an anti-HDM-2 antibody linked to 15 nm gold particles. We found multiple 6 nm- and 15 nm-labeled gold particles in approximately 1:1 ratios in layered ring-shaped structures in the pores near the cell surface suggesting that these complexes are important to the pore structure. No pores formed in the control, PNC-27-treated untransformed fibroblasts. Based on the theoretical and immuno-EM studies, we propose that the pores are lined by PNC-27 bound to HDM-2 at the membrane surface with the PNC-27 leader sequence lining the pores or by PNC-27 bound to HDM-2.

## 1. Introduction

PNC-27 and its shorter analog peptide (PNC-28) are peptides that selectively lyse the membranes of cancer cells, resulting in the necrosis of a variety of cancer cell lines and primary human tumors, but not untransformed cells [1,2,3,4,5]. PNC-27 contains residues 12–26 of the HDM-2-binding domain of p53 attached to a leader sequence (termed the membrane residency peptide or MRP) that normally induces the transport of peptides attached to it across the cell and nuclear membranes [1,2,3]. We found that this peptide induced rapid necrosis of cancer cells rather than apoptosis [1,2,3,4]. Induction of tumor cell necrosis was not induced by the isolated p53 12–26 peptide, by the leader sequence alone, or by both separate peptides together, suggesting that both domains must be covalently linked for tumor cell killing [3,4]. 

Transmission electron microscopy (TEM) of cancer cells treated with PNC-27 revealed that transmembrane pores formed in these cells [3,4]. This finding correlated with the results of the determination of the three-dimensional structure of PNC-27 by two-dimensional NMR that showed an amphipathic alpha-helix-loop-alpha-helix structure that has been found for a number of pore-forming, membrane-active polypeptides [6].

In further structural studies [7], we found that the three-dimensional structure of residues 17–26 of PNC-27 was superimposable on that determined by X-ray crystallography for the same residues of a p53 peptide (p53 residues 17–29) bound to the p53 binding domain of HDM-2 (HDM-2 residues 25–109) [8]. Since PNC-27 lacks residues 27–29, as were present in the X-ray crystal structure of the p53 19–29-HDM-2 complex, and contains p53 residues 12–16 and the entire leader sequence, that were not present in the X-ray crystal structure, the question arises as to whether PNC-27 binds to HDM-2 in a manner similar to that of the binding of the p53 peptide to HDM-2 in the X-ray crystal structure [8]. An important aspect of this question is the disposition of the leader sequence since this domain of PNC-27 has no apparent relationship to the binding of PNC-27 to HDM-2 but is critical to pore formation [3,4]. 

The selective cytotoxic pore-forming effect of PNC-27 for cancer cells is linked to elevated expression of cell surface HDM-2 compared only with low or undetectable levels in the membranes of untransformed cells [7] and its colocalization with HDM-2 in cancer cell membranes [7].

The interaction of PNC-27 with HDM-2 in cancer cell membranes, which is critical for peptide-induced tumor cell necrosis, was shown in experiments with untransformed human breast epithelial cells, which do not normally express HDM-2 in their membranes and are not susceptible to PNC-27-induced lysis [7]. Transfection of a vector encoding HDM-2 expressing a membrane localization (CAAX box) sequence into these cells resulted in extensive tumor cell necrosis when they were incubated with PNC-27. This effect was not observed in cells transfected with an empty vector or vector encoding HDM-2 with its p53 binding domain deleted but with its nuclear localization signal present, suggesting that PNC-27 binds to HDM-2 in the cancer cell membrane [7], inducing tumor cell death.

Since EM studies suggested that PNC-27 induces transmembrane pore formation uniquely in cancer cells and our further studies suggested that PNC-27 must bind to HDM-2 in the cancer cells’ membranes to induce tumor cell lysis, we now investigate, using conformational energy analysis, whether PNC-27 can form a low energy complex with the HDM-2 binding domain that is similar in structure for p53 residues 19–29 as determined in the X-ray crystal structure [8] and what the disposition of the leader sequence is in this complex. In addition, using high-resolution immuno-scanning electron microscopy, we have directly imaged PNC-27-induced transmembrane pores in cancer cell membranes and show that they contain complexes of PNC-27 with HDM-2. 

## 2. Materials and Methods

### 2.1. Materials

PNC-27 and PNC-29. PNC-27 contains a segment from human p53, residues 12–26, that is involved in the binding of p53 to HDM-2 connected to a membrane penetrating sequence called the membrane residency peptide (MRP). The sequence is H-Pro-Pro-Leu-Ser-Gln-Glu-Thr-Phe-Ser-Asp-Leu-Trp-Lys-Leu-Leu-*Lys-Lys-Trp-Lys-Met-Arg-Arg-Asn-Gln-Phe-Trp-Val-Lys-Val-Gln-Arg-Gly-OH*. The MRP is shown in italics. This peptide (1 g) was synthesized using solid-phase methods (Shaanxi Zhongbang Pharma-Tech Corp., NanguanZhengjie, Xi’an, China) and was >95% pure by HPLC and mass spectrographic analysis. Similarly, the negative control peptide, PNC-29 [1,2,4], containing the X13 peptide from cytochrome P450 (bold) attached to the MRP (italics), H-Met-Pro-Phe-Ser-Thr-Gly-Lys-Arg-Ile-Met-Leu-Gly-Glu-*Lys-Lys-Trp-Lys-Met-Arg-Arg-Asn-Gln-Phe-Trp-Val-Lys-Val-Gln-Arg-Gly-OH*, was likewise synthesized by solid-phase methods (Biopeptides, San Diego, CA, USA) and was >95% pure by the same techniques.

Streptolysin O. This was purchased from Sigma (St. Louis, MO, USA) and used directly.

Cells. MIA-PaCa-2 (human pancreatic carcinoma) and A2058 (human melanoma) cells and untransformed human fibroblasts (AG13145) were purchased from the American Type Culture Collection (ATCC) (Manassas, VA, USA) and placed in the appropriate media as described previously [1]. BMRPA1.TUC-3 (rat pancreatic cancer) cells were grown in our laboratory using methods described previously [2,3,4,7].

### 2.2. Methods

Predicted Structure of PNC-27 Bound to the HDM-2 p53 Peptide Binding Domain Residues 25–109. We superimposed the atoms of our 2D-NMR-determined structure for PNC-27 [6] for residues 17–26 of the p53 HDM-2-binding segment of PNC-27 on the corresponding coordinates for the atoms, from the X-ray crystal structure of the p53 peptide residues 17–29 complexed with residues 25–109 of HDM-2 [5] as deposited in the Protein Data Bank as 1YCR, using PyMol (PyMol Molecular Graphics System Web Site). Unlike in the previous study [6], we performed the superposition using the whole peptide. After performing the supposition of the PNC-27 structure on the corresponding structure of the p53 peptide in the HDM-2 binding site, we removed the 17–29 peptide so that the complex of the HDM-2 structure with our least-squares best-fit structure for PNC-27 was the starting structure for energy minimization using Maestro (Schrodinger Release 2016-2, Version 10.6, New York, NY, USA). The latter process was carried out using the method of steepest descents allowing the atoms of both the PNC-27 peptide and HDM-2 to change [9]. After energy convergence, we then superimposed the coordinates for residues 17–26 of the resulting energy-minimized structure with those of the corresponding residues of the X-ray structure for the 17–29 residue peptide bound to HDM-2 to determine if the structure of the residues of PNC-27 was still superimposable. 

#### Transmission Electron Microscopy (TEM)

TEM-Cells. Cells (1 × 10^6^) were grown in 6-well dishes overnight; the spent medium was removed, and the cells were washed with PBS and treated at 37 °C with PNC-27 at pre-determined concentrations. After 10 min of PNC-27 treatment, the cells were washed and fixed in 3% buffered paraformaldehyde supplemented with 1% glutaraldehyde for 2 h at which time they were scraped into PBS and centrifuged into a pellet. The pellet was embedded in 6% agar in PBS followed by osmication and dehydration through sequential passages in increasing concentrations of ethanol (50–100%). The pellet was embedded in 50% resin for 1 h followed by 100% resin embedment overnight. Samples were processed for TEM by preparing 60 nm thin sections that were stained with uranyl acetate and examined in a Zeiss EM10 TEM.

Immunogold TEM. Cells (1 × 10^6^) were grown in 6-well TCD overnight when spent medium was removed; the cells were then washed with PBS and treated at 37 °C with PNC-27 in PBS. After 10 min, the cells were washed and fixed in 3% buffered paraformaldehyde supplemented with 0.1% glutaraldehyde for 1.5 h. After extensive washing, quenching with glycine and NaBH4, the cells were incubated overnight at 4 °C with anti-p53 mAb clone DO-1 that recognizes p53 residues 11–25, overlapping with the PNC-27 p53 residues 12–26 domain (Active Motif, Carlsbad, CA, USA) or monoclonal rabbit anti-HDM -2 (Pierce, Rockford, IL, USA), each at 5 μg/mL. After removal of unbound Ab and washing with PBS, the cells were incubated for 6 h with 6 nm gold-conjugated GαM F(ab’)_2_ (Abcam, Cambridge, MA, USA) or 15 nm gold-conjugated GαR F(ab’)_2_ (Abcam). After extensive washing, cells were post-fixed in 1% glutaraldehyde in cacodylate buffer (0.113 M, pH7.2) overnight when the fixed cells were scraped into PBS, centrifuged into a pellet, washed in cacodylate buffer, and post-fixed in 1% osmium tetraoxide. Following embedding and sectioning as described above, the thin sections were stained with uranyl acetate and examined in a Zeiss EM10 TEM. 

Streptolysin Control. In a separate set of control experiments, A2058 human melanoma cells were treated with streptolysin O (Santa Cruz Biotechnology, Dallas, TX, USA), Rabbit IgG against SLO (RαSLO IgG) (Santa Cruz Biotechnology) and secondary antibody, i.e., 6 nm gold particles conjugated to Goat IgG against RIgG (gold-GαRIgG) was obtained from Electron Microscopic Sciences (EMD, Ft. Washington, PA, USA). In a prior series of preliminary dose-response experiments over a 30 min incubation period at RT, 10 hemolytic units (HU)/mL were established as the LD_50_ in A2058 cells. After 10 min of SLO-treatment at room temperature, unbound SLO was washed off with ice-cold PBS and the cells were fixed and processed as described above. The cells were then successively stained with RαSLO IgG and 6 nm gold-derivatized GαR IgG, post-fixed in 1% glutaraldehyde in cacodylate buffer, scraped and processed for TEM on a Zeiss EM10 TEM as described above. Photography was performed using an automated digital camera providing a scale bar for each picture that accompanied the preparation of the present photographs and defines, in any comparisons, the correct sizes of cells and cellular organelles.

Scanning Electron Microscopy (SEM). MIA-PaCa-2 cells were grown on glass cover slips and treated with PNC-27 (150 μg/mL) for 3–5 min at 37 °C. Control cells were left untreated in the same buffer conditions. At the end of the incubation period, the cells were fixed in 3% glutaraldehyde in 0.113 M cacodylate buffer (pH7.4) at 37 °C in a water bath for 15 to 30 min, followed by overnight fixation at room temperature. The cells were then immuno-gold stained with antibodies against PNC-27 and HDM2 as described for TEM immuno-gold experiments. The cells were then dehydrated by moving each cover slip through a series of increasing alcohol concentrations from 50% to 90% (2 incubations each for 5 min) and 100% (2 incubations, 10 min each). The cover slips with the dehydrated cells were then mounted on metal stubs, platinum sputter-coated and viewed in a LEO 1550 SEM, capable of detecting high back-scattering of the gold particles, equipped with an automated digital camera that inserted scale bars according to the magnifications used during photographic recording of the results. Identical procedures were carried out for untransformed AG-13145 human fibroblasts.

Temperature Dependence of PNC-27 Tumor Cell Cytotoxicity. A series of individual 96-well tissue culture dishes (TCDs) were prepared and cells (5000/well) were seeded into each well. To precisely control the incubation temperatures, the TCDs with the cells were floated in water baths set to 17 °C and 37 °C. The temperatures in the wells were continuously controlled by thermometers placed directly into a buffer-containing well of each TCD. Once the medium remained stable at the preset temperature, the original culture medium was removed and PBS containing the concentrations of peptide indicated was added. After 30 min incubation at the predetermined temperature, the cytotoxic effect of PNC-27 was calculated in percent from the amount of released LDH (cell supernatant) in the wells, and the amount of LDH remaining in the cells (cell lysates) as described previously (2–5; 7). All experiments (n = 3) were carried out using triplicate sets of wells. Three cell lines were employed in this study: Rat pancreatic cancer BMRPA1.TUC-3 cells, Hu-melanoma (A2058) cells, and Hu-pancreatic cancer MIA PaCa-2 cells. 

Cytotoxicity Studies Involving Incubation at 17 °C followed by incubation and Cytotoxicity Assay at 37 °C. From BMRPA1-TUC-3 pancreatic cancer cells that had been incubated with PNC-27 at 17 °C for 30 min, the supernatant was removed, the cells were washed with PBS (17 °C), which was followed by re-incubation in only PBS (37 °C) at 37 °C, while another set of cells in triplicate continued incubation at 17 °C. Thirty minutes later, cytotoxicity was measured, as described previously [2,3,4,5,7], in all wells. 

## 3. Results

**PNC-27 Is Accommodated in the HDM-2 Binding Site.** PNC-27 is a peptide that contains residues 12–26 of the p53 protein that are involved in the binding of p53 to HDM-2 attached to the leader sequence on its carboxyl terminal end. Since the residues of the p53 peptide domain that make contact with residues in the binding site of HDM-2 are near to the carboxyl terminal end of this peptide domain [8], there is the possibility that the leader sequence might disrupt interactions between the peptide and the HDM-2 residues involved in binding. 

To explore whether the leader sequence can be accommodated in the HDM-2 binding site, we superimposed the structure of residues 17–26 of PNC-27 that we determined by 2-D NMR (6) on that for the corresponding residues for the p53 17–29 peptide bound to residues 25–109 of HDM-2 as determined in the X-ray crystallographic study [8] and minimized the conformational energy of the resulting complex [9,10]. 

**Figure 1:**
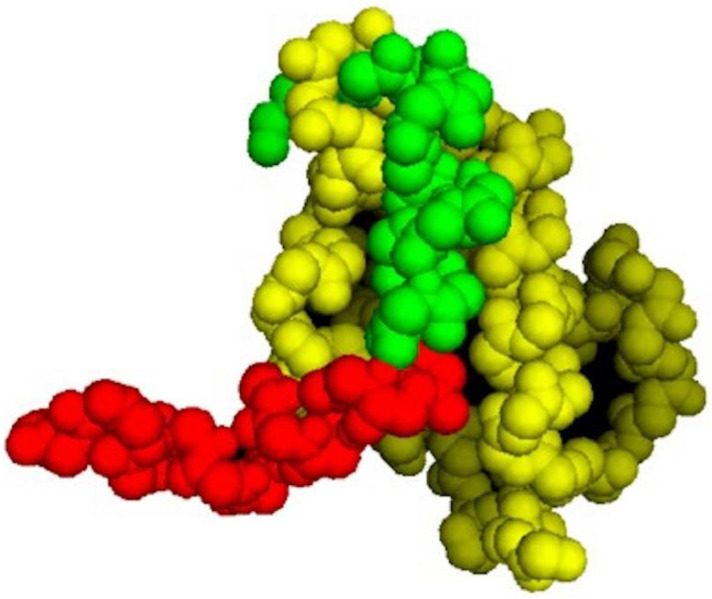
**Energy-minimized structure of PNC-27 bound to the p53-binding site of HDM-2.** Residues 25–109 from the X-ray crystal structure of the HDM-2 binding site. Coordinates for this structure are available as 1YCR in the Protein Data Base. Colors: green PNC-27 p53 residues 12–26 (residues 1–15 in PNC-27); red: membrane residency peptide (MRP) of PNC-27 (residues 16–32 in PNC-27); and yellow, HDM-2 p53 binding domain.

As shown in Figure 1, the p53 segment of PNC-27 in the energy-minimized structure (green residues) is buried in the HDM-2 binding site (yellow residues in Figure 1). Comparison of the structure of PNC-27 residues 17–26 from the energy-minimized structure with that of the corresponding residues of the 17–29 p53 peptide in the X-ray structure resulted in an RMS deviation of 0.7 Å, suggesting that PNC-27 can bind to HDM-2 in a manner that is close to that for the peptide in the X-ray crystallographic structure. These results further suggest that the MRP (red residues) does not disrupt the critical interactions between PNC-27 and HDM-2. Furthermore, as shown in Figure 1, the energy-minimized structure is accommodated in the HDM-2 binding site such that the leader sequence protrudes away from the complex into the solvent (Figure 1). This finding may imply that the MRP is free to be involved in pore formation as discussed further below.

**Figure 2:**
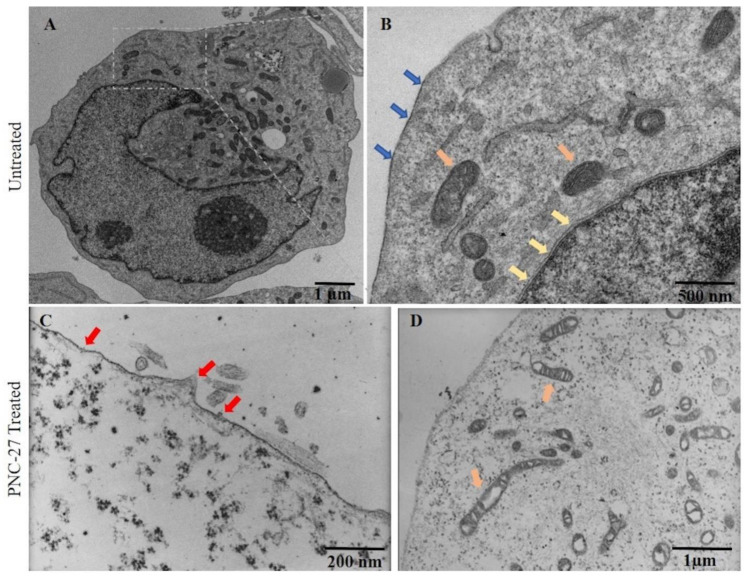
**TEM of cancer cells treated with PNC-27 shows disrupted plasma membrane and mitochondria.** MIAPaCa-2 cancer cells were either left untreated (**A**): low magnification; (**B**): high magnification) or treated with PNC-27 for 3 min; (**C**): membrane view at high magnification; (**D**): cytoplasmic view at high magnification. In (**B**), blue arrows show plasma membrane integrity; orange arrows show mitochondria and yellow arrows show nuclear membrane. In (**C**), red arrows show site of pores in the membrane.

**PNC-27 Induction of Transmembrane Pores in Cancer Cells.** In prior publications [3,4], we presented the results of TEM showing pores that formed in cancer cell membranes even after short exposures of the MCF-7 breast cancer cells to PNC-27 [3] and MIA-PaCa-2 human pancreatic cancer cells to PNC-28. Figure 2 shows the effects of PNC-27 on MIA-PaCa-2 cells after 10 min of incubation. Both panels A and B of this figure show untreated MIA-PaCa-2 cells with intact plasma (blue arrows) and nuclear (yellow arrows) membranes. Panels C and D show that, after several minutes of treatment of the cells with PNC-27, pores form in the plasma membrane (red arrows, Panel C) and vacuoles form in mitochondria (orange arrows, Panel D). As controls, these cells were incubated with a control peptide, PNC-29, known to have no effect on either cancer or normal cells [3,4], and, as shown in Supplementary Materials, Figure S1 (Panels A and B), the cells remained intact. As a further control (Panels C and D in Supplementary Materials, Figure S1), untransformed human fibroblasts (AG13145) and untransformed pancreatic acinar BMRPA-1 cells were incubated with PNC-27 and likewise remained intact. Thus, PNC-27 induces trans-membrane pore formation in MIA-PaCa-2 cancer cells but not in normal (untransformed cells).

**PNC-27 Is Present in Pores in Cancer Cell Membranes.** To determine whether PNC-27 occurs in transmembrane pores in cancer cells, we utilized antibodies linked to homogeneous-sized gold particles. This technique has been used to show that membrane-lysing proteins, such as streptolysin O (SLO), are directly involved in transmembrane pore formation [11]. 

To evaluate whether there are similarities in pore structures induced by PNC-27 to that of known pore-forming agents (i.e., SLO), we incubated MIA-PaCa-2 cells with SLO (10 hemolytic units/mL) for 10 min, washed, and then incubated the treated cells with rabbit IgG against SLO, followed by incubation with 6 nm diameter gold-labeled goat anti-rabbit IgG. The cells were washed and fixed for TEM. Results are shown in the Supplementary Materials, Figure S2. Supplementary Materials Figure S2A shows a low power view of a MIA-PaCa-2 cell whose membrane has been disrupted upon treatment with SLO labeled as small black dots representing gold-labeled anti-SLO antibody. At higher magnification (Supplementary Materials, Figure S2B, showing the region surrounded by the dashed box), discrete ring-shaped pores can be seen. The pore structure is surrounded by gold-labeled spheres (black dots), labeling SLO in a manner very similar to the results of previous studies [11]. 

**Figure 3:**
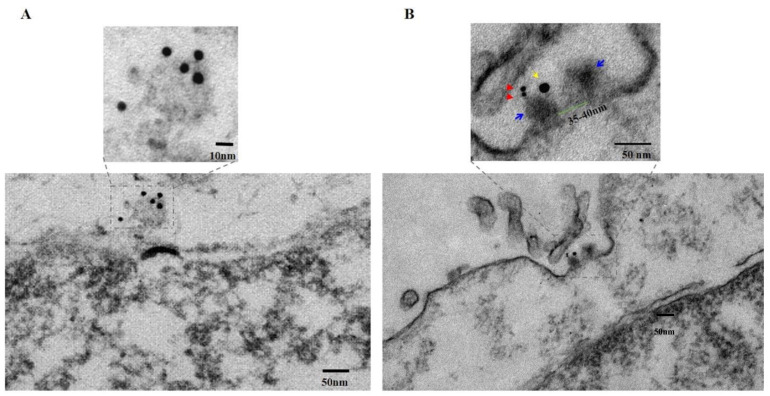
**TEM: Pore formation and co-localization of PNC-27 and HDM2 on the plasma membrane of cancer cells.** Immuno-transmission electron micrograph of PNC-27-treated human pancreatic cancer MIAPaCa-2 cells (100 μg/mL for 10 min) showing: (**A**): **Lower** panel: disrupted membrane with a pore on the top middle of the figure indicated by the dashed box. The black dots are gold-labeled PNC-27 molecules that surround the pore. (**A**): **Upper** Panel: higher magnification of the pore structure in the lower part of the figure. Pore structures identified by anti-PNC-27 antibody labeled by 6 nm gold-labeled secondary antibody. (**B**): **Lower** Panel: longitudinal section of the membrane of a MIAPaCa-2 cell treated with PNC-27 (100 μg/mL [25 uM] for 3 min) and labeled with 6 nm gold particles attached to the anti-PNC-27 antibody system and 15 nm gold particles attached to the anti-HDM-2 antibody system. Longitudinal-section of a transmembrane pore is shown surrounded by the drawn dashed rectangle. The bar on the lower right corner is 50 nm. (**B**): **Upper** Panel: higher magnification of the pore seen in the lower figure. A longitudinal section of a pore stained for 6 nm gold-labeled PNC-27 labeled (red arrows) as in part A and with anti-HDM-2 antibody labeled by 15 nm gold-labeled secondary antibodies (yellow arrow). The blue arrows point to the “bulky head” structures that are the top part of the pore, extending outwards from the pore.

Next, using similar methodology, we treated MIA-PaCa-2 cells with PNC-27 in PBS. After 10 min at 37 °C, cells were washed and incubated with mouse anti-PNC-27 monoclonal antibody specific for p53 component (12–26 segment), followed by incubation with 6 nm gold-conjugated GαM F(ab’)_2_. After washing off unbound antibodies, the cells were fixed and processed for TEM as described. As shown in Figure 3A, a well-defined ring structure similar to that observed in cells treated with SLO (Supplementary Materials, Figure S2) appears in the lysing cell. This structure is composed of PNC-27, which is identified by indirect immune-gold staining with monoclonal antibody specific for p53 portion of PNC-27. Decorating the ring structure are 6 nm gold particles identifying PNC-27. This finding suggests that PNC-27 occurs in pores in the cancer cell membrane.

**Figure 4:**
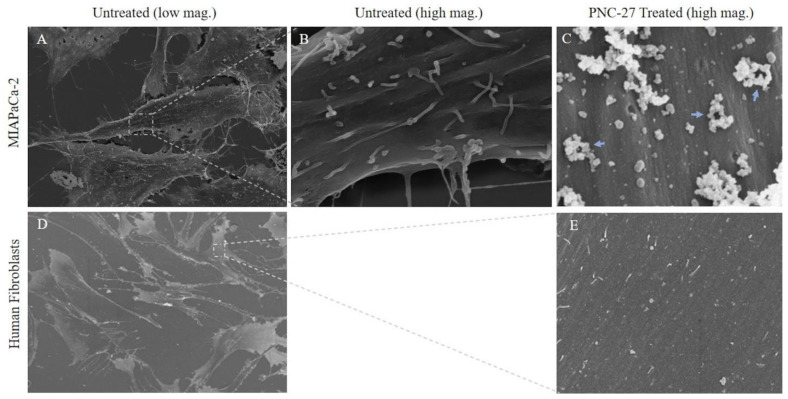
**SEM: Cancer vs. untransformed cells treated with PNC-27.** (**A**): Scanning electron micrograph of untreated MIA-PaCa-2 cells. (**B**): High power scanning electron micrograph of the membrane of an untreated MIA-PaCa-2 cell in (**A**). (**C**): Scanning electron micrograph of the membrane of a typical MIA-PaCa-2 cell from cells that were treated with PNC-27 for 10 min. The holes are transmembrane pores surrounded by spherical bodies that protrude out from the membrane into the external environment (blue arrows). (**D**): Scanning electron micrograph of untreated untransformed human fibroblasts (AG-13145). (**E**): Scanning electron micrograph of the membrane of a fibroblast treated with PNC-27 showing no pores. low mag. (×5000); high mag. (×100,000).

**PNC-27-HDM-2 Complexes occur in Pores in the Plasma Membranes of Cancer Cells.** To determine further if PNC-27 occurs in complexes with HDM-2 in transmembrane pores, we performed the same experiment as in Figure 3A, except that the PNC-27 treatment was reduced to 3 min and we also labeled HDM-2 with a 15 nm gold-labeled antibody. Figure 3B (lower panel) shows a pore through the cancer cell membrane that has been cut in longitudinal section. As seen in the upper panel of Figure 3B, a high power view of this section of the region enclosed in the dashed box in the lower panel of Figure 3B, the continuity of the membrane is disrupted by a transmembrane pore-like structure, measured with an inside diameter of 35–40 nm (the channel; green bar) and outside diameter of 80–90 nm (the pore complex; upper panel, Figure 3B). The two walls of the pore complex appear to have penetrated through both layers of the lipid bi-layer while extending beyond the extracellular surface membrane by 8–10 nm, ending in two “bulky heads” (indicated with blue arrows, upper panel, Figure 3B) that, in fact, protrude into the extracellular space. These results are compatible with those shown in Figure 4C discussed below. One of the “bulky” heads is stained with two closely apposed 6 nm gold particles (red arrows, upper panel, Figure 3B), indicating the presence of PNC-27 protein antigen while nearby, within <17 nm distance, on the same “bulky head” is located one 15 nm gold particle (yellow arrow, upper panel, Figure 3B), indicating the presence of HDM-2 in the same complex as the PNC-27 peptide.

By applying the specificity of the immunostaining reaction to the structure of the pore, it can be stated that the two heads likely represent part of the external rim of the pore formed by the assembly of the PNC-27-HDM2 complexes entering into the pore complexes. As noted above, the longitudinal section of the PNC-27-HDM2 pore complex is very similar to that of channels of SLO [11]. The morphological similarity of SLO pores and PNC-27-HDM2 pore complexes, together with the fatal outcome for cells whose plasma membranes have been penetrated and made leaky by the insertion of SLO pores, suggests a similar functional consequence to the formation of PNC-27-HDM-2 pore complexes as the cause of rapid cancer cell necrosis induced by PNC-27. 

**Three-Dimensional Structure of PNC-27-Induced Transmembrane Pores Containing PNC-27-HDM-2 Complexes.** To achieve a better view of the pore structure and its relation to the cell surface topography and the participating molecules (PNC-27 and HDM-2), we performed scanning (three-dimensional) EM (SEM) on cancer cells treated with PNC-27 to examine the structure of these pores not visible in the TEM studies. In these experiments, MIA-PaCa-2 cells were treated with PNC-27 for 3 min after which they were processed for SEM. 

Figure 4 shows untreated MIA-PaCa-2 cells (Figure 4A) and a high-power view of the membrane of one of these cells (Figure 4B). Figure 4C shows the membrane of one of the MIA-PaCa-2 cells treated with PNC-27. The membrane is covered with pores surrounded by well-defined spherical structures (blue arrows) some of which appear to protrude from the membrane into the external environment. 

As a control for these studies, we treated the untransformed cell line, AG-13145 human skin fibroblasts, with PNC-27 under identical conditions to those used for MIA-PaCa-2 cells. As shown in Figure 4D (untreated fibroblasts) and 4E (fibroblasts treated with PNC-27), these cells, under SEM, were found to have neither pores nor spherical bodies surrounding pores as found in Figure 4C. This finding confirms that the membrane of untransformed fibroblasts remains smooth after being treated with PNC-27 and does not contain any pores or pores surrounded by spherical structures seen in the cancer cells. 

To determine the spatial relationship of PNC-27 and HDM-2 in the structure of PNC-27-induced pores, we obtained high-resolution back-scattered SEM with gold immunostaining, as described for the experiments performed for Figure 3B. The objective was to obtain 3-D images of the cancer cell’s surface membrane and the pore structures. 

**Figure 5:**
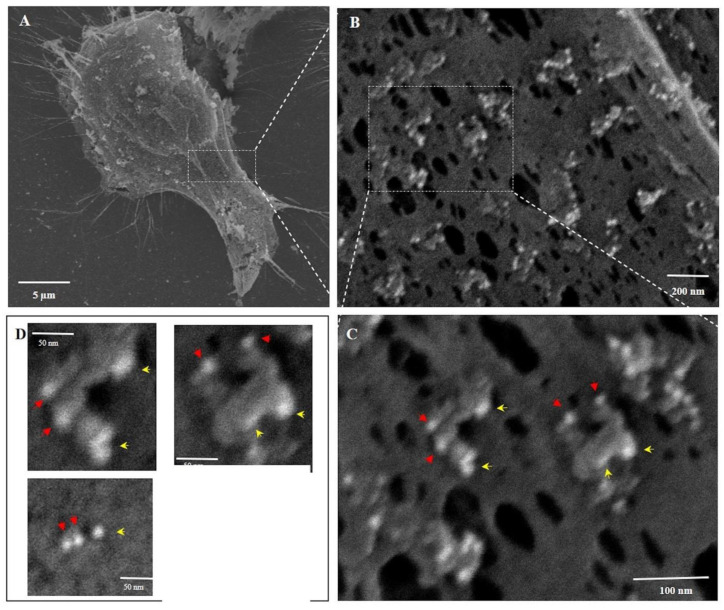
**SEM: PNC-27-induced pores in MIAPaCa-2 cells identified by immuno-scanning electron microscopy (high backscatter).** These cells were treated with PNC-27 (150 μg/mL [37.5 uM]) for 3 min. Panel (**A**): MIA-PaCa-2 cell showing granular structures on the surface of the cell; Panel B shows pores surrounded by protruding white-colored spherical structures which are the gold particles. Panel (**C**): high magnification of the structures in the dashed box in Panel (**B**). These structures are identified as containing 6 nm gold (red arrowheads) labeling PNC-27 and 15 nm gold particles (yellow arrowheads) labeling HDM-2. All of these spherical structures are seen to contain PNC-27-HDM-2 complexes. Panel (**D**): Upper two sub-panels show details of two pores shown in the upper right panel; the lower right sub-panel shows measurements of pore diameters and gold particle sizes. The lower left sub-panel shows a pore with 2 PNC-27 molecules and one HDM-2 surrounding it.

Figure 5 shows the plasma membrane of PNC-27-treated MiaPaCa-2 cells at four successively increasing magnifications. In Panel A, the surface membrane appears rough and stippled as opposed to the untreated cells (Figure 4A). At higher magnification, as shown in Figure 5B–D, numerous see-through pore-like channels are seen distributed over the plasma membrane. These pore-like channels are surrounded by limiting rings, which appear as donuts or partial donuts, and appear to be projecting out from the membrane level, constituting the “bulky heads” that project beyond the cancer cell membrane shown in Figure 3B. 

Although the appearance of the pores in Figure 5B–D is similar to those shown in Figure 4C, the white-colored appearance of the spherical structures in Figure 5 is due to the high back-scattering of light by the gold particles. If the gold atoms attached to the two antibody systems labeling PNC-27 and HDM-2 had not been present, the white spherical structures surrounding the pores in Figure 5 would have a gray to black color, making it impossible to identify these structures. We have confirmed this conclusion in control experiments in which cells treated with PNC-27 were then incubated only with secondary antibodies and then processed for SEM. Therefore, we conclude that the spherical structures shown in Figure 4C contain complexes of PNC-27 and HDM-2. 

Higher magnification of the ring structures shown in Figure 5B is shown in Figure 5C where two typical surrounding ring structures are seen to be composed of gold-containing particles that are 6 nm (red arrows) and 15 nm (yellow arrows) in diameter, indicating the presence of PNC-27 and HDM-2, respectively; these appear to be formed as 1:1 complexes. Virtually all of the donut-like structures in Figure 5C are decorated with these complexes surrounding the pores. These donut-like rings enclose transmembrane pores that, when measured, have inside diameters of similar sizes of 28–44 nm with an average diameter of 34.5 ± 5.6 nm (*n* = 100), similar to the inner pore diameter found in Figure 3B.

The two typical pore structures in Figure 5C are shown at higher magnification in the two upper panels of Figure 5D. As shown in the lower right panel of Figure 5D, the pore space itself was found to have a diameter of 37.7 nm. This pore was surrounded by two PNC-27 and one HDM-2 gold-labeled particle (lower left panel in Figure 5D). The lower left panel in 5D shows examples of pores that appear to not follow the 1:1 ratio of peptide to HMD2, which may be explained by the spatial limitations and positioning of the primary, secondary, and the gold particles attached to secondary antibodies surrounding the pore. The presence of 4–8 IgG antibody molecules as well as large gold particles may be a limiting factor in spatial availability that can lead to dissociation of these complexes during SEM processing. To help confirm that the spherical particles contain gold-labeled proteins, we measured the diameter of these particles on the EM images and found that they are 7.69 nm for the nominal 6 nm gold particle and 15.05 nm for the 15 nm gold particles. 

The unlabeled holes in the PNC-27-treated membrane of these cancer cells may have been caused by dissociation of the pore structure from the membrane or may have resulted secondarily from disruption of the plasma membrane due to the inability of the cells to maintain their fluid and ion balance and energy production. Since PNC-27-HDM-2 complexes surround most of the pores, these observations provide strong support for the notion that PNC-27 molecules inserted into the plasma membrane of cancer cells bind to HDM-2, and the resulting complexes associate to form structured transmembrane pores. 

**Steps in the Formation of Transmembrane Pores.** It has been proposed for pore-forming proteins, such as SLO, that the process of pore formation involves two steps: one in which the protein binds to the membrane and, then in a second step, the protein diffuses through the lipid membrane, forming the transmembrane pores [11,12,13]. The first binding step appears to be a temperature-independent process (within about 4–35 °C), while the diffusion step appears to be strongly temperature-dependent over the same temperature range [11,12,13]. The temperature dependence is likely due to decreased lipid mobility as the temperature is lowered [11,12,13]. The first step may involve major changes in the domain structure of the protein as has been modeled for pneumolysin [14,15]. In this process, protein molecules may associate with one another independently of the membrane and form isolated pores or “arcs” in the cell membrane [14,15]. 

**Figure 6:**
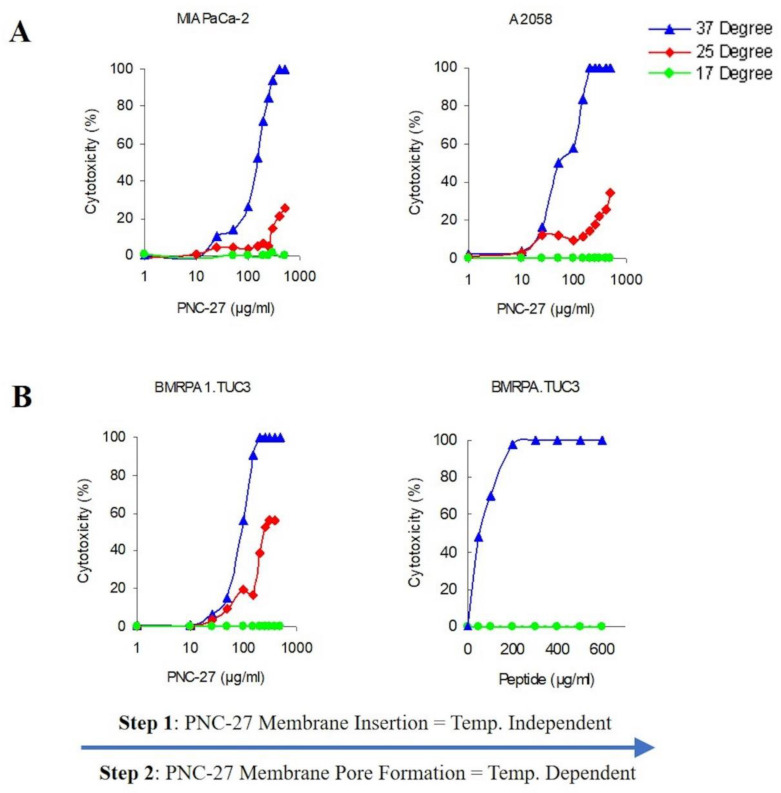
**PNC-27 cytotoxicity effect is temperature-dependent.** (**A**): Dose-response curves for two different cancer cell lines as labeled in the figure: MIA-PaCa-2 and A2058 at 3 different temperatures: 37 °C (blue), 25 °C (red), and 17 °C (green). LDH Cytotoxicity assay was performed to evaluate cell killing. Each point represents the average ± SEM of 3 experiments. (**B**): Dose-response curves for BMRPA1.TUC-3 cells treated with PNC-27. Cells were incubated with different concentrations of PNC-27 at 17 °C. LDH assays for membranolysis were performed for each incubation mixture (green). The cells were then washed to remove any unbound PNC-27, re-plated, brought to 37 °C for 30 min, and re-assayed (blue). Each point represents the average ± SEM of 3 experiments. The triangles (blue), diamonds (red), and filled circles (green) represent the mean at each peptide dose. The standard deviations cannot be seen due to their low values. All doses of PNC-27 are given in each plot as ug/mL. To convert to micromolar concentration units, multiply each dose in the figure by the factor 0.25.

Therefore, to further define the process of pore formation induced by PNC-27, we studied the temperature dependence of its ability to bind to HDM-2 and induce pore formation in the light of these previous studies. In these experiments, we assayed the release of LDH as a measure of pore formation since this correlates with tumor cell killing and transmembrane pore formation. Figure 6A shows the temperature dependence of PNC-27-induced pore formation for two different cell lines MIA-PaCa-2 (pancreatic cancer) and A2058 (melanoma). As can be seen in Figure 6A, there is a strong temperature dependence for induction of pore formation as observed by the release of LDH from the treated cells. Dose-response experiments at 37 °C reach 100 percent tumor cell killing at concentrations above 100 μg/mL as measured by LDH release. Lowering the temperature by just 12 °C to 25 °C results in a large decrease in cell killing, and, at 17 °C, there is virtually no cell killing (Figure 6A). 

If the above-described model for pore formation holds, PNC-27 should be bound to HDM-2 but the individual complexes would be unable to aggregate into pores at the low temperatures. To test this hypothesis, we incubated rodent pancreatic cancer BMRPA1.TUC-3 cells at 17 °C with PNC-27 at the same concentrations as used for the temperature experiments shown in Figure 6A, and washed out the excess peptide. The cells were then warmed to 37 °C over 30 min. The results are shown in Figure 6B. Cell killing was enhanced as the PNC-27 molecules had already been inserted into the membrane of the cells, and by increasing the temperature to 37 °C, the monomeric peptide molecules were then able to freely move within the membrane and aggregate to form the pores in a shorter time period. These results suggest that monomeric PNC-27 molecules, like SLO, bind to its target in a relatively temperature-independent manner and then form pores by diffusion of the PNC-27-HDM-2 complexes to form aggregates in the cell membrane in a temperature-dependent process. 

## 4. Discussion

**PNC-27 Induces Transmembrane Pores in Cancer Cells that Contain PNC-27-HDM-2 Complexes.** The results shown in Figure 2, Figure 3, Figure 4 and Figure 5 suggest that PNC-27 induces the formation of transmembrane pores lined at the outer membrane surface with PNC-27-HDM-2 complexes. Many of these complexes appear to protrude beyond the membrane surface into the extracellular environment, i.e., the “bulky heads”, seen in the TEM experiments shown in Figure 3B, as well as the spherically shaped particles surrounding the pores in the membrane seen in the SEM experiments of Figure 4 and Figure 5. Although, due to different angles of cutting cell samples, it is not possible to establish the exact stoichiometry of the complexes, Figure 5 suggests that PNC-27 and HDM-2 form approximately one to one complexes. 

**Relationship of PNC-27-HDM-2 Complexes to Pore Structure.** In a recent study [16], it was found that PNC-27 induces tumor cell necrosis of a number of human and mouse acute myelogenous leukemia (AML) cells. All of these cells expressed HDM-2 in their cell membranes with which PNC-27 was found to co-localize as we found in our prior experiments on human solid tissue tumors [7]. Transmission electron micrographic studies of these cells that had been treated with PNC-27 were found to have transmembrane pores. In separate experiments [16] in the same study, PNC-27 was found to induce interaction of HDM-2 with the membrane protein cadherin resulting in its ubiquitination and cleavage. Transfection of these cells with siRNAs against expression of cadherin resulted in markedly decreased cell killing induced by PNC-27 leading to the conclusions that PNC-27-induced pore formation was caused by HDM-2-promoted degradation of cadherin and that PNC-27-HDM-2 complexes were not involved in the formation of pores. 

Our present results on solid tissue tumors suggest that the pores induced by PNC-27 in the solid tissue cancer cells, as revealed by high-resolution images of the structure of the pores, are lined with PNC-27-HDM-2 complexes and are essential to pore structure as we discuss further below. 

**Possible Structure of Pores. PNC-27 adopts an amphipathic alpha-helix-loop-alpha-helix conformation in solution** [6]. *This type* of structure occurs in a number of membrane-active peptides such as mellitin [17] and magainin [18]. A model for transmembrane pore formation induced by amphipathic peptides has been proposed [19] in which the amphipathic peptide forms pores in several stages. In the first stage, the peptide lies along the outer cell membrane wherein the positively charged peptide residues (such as those that occur in the MRP) interact with phosphate negative charges in the phospholipids of the membrane. In a second step, the peptide inserts into the membrane such that the hydrophobic residues interact with lipids in the lipid bilayer while the charged and polar residues form intermolecular complexes with one another lining the interior of the pore. In this step, parts or all of the peptide can undergo a helix-to-extended conformation to span the membrane [19].

Though PNC-27 is an amphipathic peptide, it is clear that it forms complexes with HDM-2 in the transmembrane pores. Furthermore, we have found that, in normal pancreatic acinar (BMRPA1) cells, PNC-27 traverses the membrane and localizes to the nucleus [4]. No pores form in these or other untransformed cells treated with PNC-27 (see Figure 4E showing no pores in untransformed fibroblasts treated with PNC-27). Thus, a prerequisite for PNC-27-induced transmembrane pore formation is that it complexes with membrane-bound HDM-2. In addition, most of the hydrophobic face of the PNC-27 that is composed of residues 6–15 (corresponding to residues 17–26 of p53) in the HDM-2 binding domain, from our structure calculations, is bound deeply within the binding pocket of HDM-2 (Figure 1) and would not be available to interact with membrane bilayer lipids.

These considerations raise the question as to the possible arrangements of the PNC-27-HDM-2 complexes in the pore. In particular, do PNC-27-HDM-2 complexes line the pores from the surface of the membrane down to the cytosolic side of the membrane? The existence of PNC-27-HDM-2 complexes contained within the membrane seems unlikely since this complex would have to exist in a largely hydrophobic lipid bilayer environment. 

If PNC-27-HDM-2 complexes form near the surface of the cancer cell membrane, there are several possibilities for the structure of the resulting pores. One of these would be the function of HDM-2 as an anchor for binding to PNC-27. This initial complex would serve as a surface for initiating the coalescence of other PNC-27 molecules such that each pore would be lined with transmembrane PNC-27 molecules. Our results are inconsistent with such a model since this arrangement would involve the binding of one HDM-2 molecule with multiple PNC-27 molecules. In contrast, we observe approximately 1:1 complexes between these molecules in the pores (Figure 5C,D). Furthermore, as noted above, PNC-27, unbound to HDM-2, diffuses through the cell membrane [4] into the cytosol, making this model unlikely.

**Figure 7:**
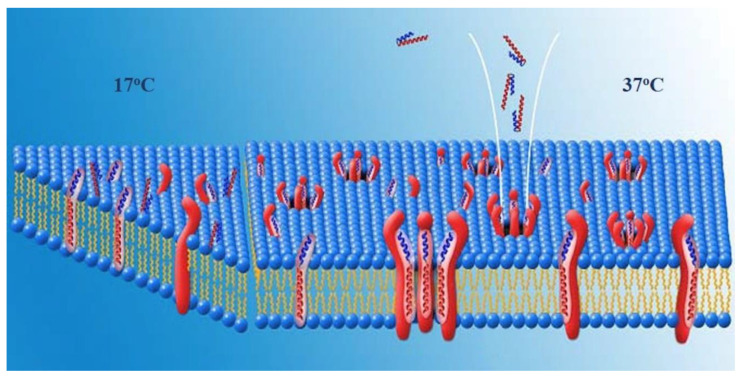
**Model for formation of transmembrane pores induced by PNC-27.** This figure is divided into two parts: on the (**left**), a model is shown as a triangular pie-shaped section of the cancer cell membrane, in which PNC-27, shown as the blue (the HDM-2-binding portion) and red (MRP or leader sequence) bent α-helical structure, binds to HDM-2, shown as the large red transmembrane molecules at low temperature (17 °C), but the complex does not form pores. At higher temperature (37 °C), as shown in the center and (**right**) of the figure, these PNC-27-HDM-2 complexes coalesce to form transmembrane pores such as the one in the middle of the figure. In this figure, HDM-2 is shown as spanning the entire membrane. Alternatively, PNC-27 may bind directly to HDM-2 near the cell surface and HDM-2 may not extend deeply into membrane, but rather the MRP may extend through the membrane in an extended conformation such that the pores would be lined by the MRP.

A second possible arrangement of the complexes would be one in which HDM-2 is a transmembrane protein and the pores are lined by HDM-2-PNC-27 complexes through the cell membrane. In this arrangement, residues of both PNC-27 and HDM-2, as illustrated in Figure 7, would line the pore. In the first temperature-independent step (labeled 17 °C in Figure 7), PNC-27 would bind to HDM-2, shown as a transmembrane protein as illustrated on the left of Figure 7. In a second, temperature-dependent step (labeled 37oc in Figure 7), shown in the middle and right side of Figure 7, PNC-27-HDM-2 complexes would associate in the membrane to form pores lined by transmembrane HDM-2-PNC-27 complexes. 

**Possible Role of the MRP in PNC-27.** Alternatively, if HDM-2 is expressed on the cell surface and does not traverse the cell membrane, complexes of HDM-2 and PNC-27 could coalesce such that the MRP (leader sequence) (red residues in Figure 1) would span the membrane and line the pores. The energy-minimized structure in Figure 1 shows that the MRP (red structure in Figure 1) is not involved in binding to HDM-2, projecting away from the complex, and appears to be free to interact with other molecules. This conclusion must be qualified by the consideration that only the p53-binding domain of HDM-2, residues 25–109, bound to a p53 peptide has been subjected to X-ray crystallographic analysis (13). Full-length HDM-2 has 491 amino acids. The location of the MRP in full-length HDM-2 is therefore unknown. However, if the MRP segment does project away from the complex, it may be in a position to line the pores.

In this model, if the MRP retains its α-helical structure, the maximal length of the MRP sequence (residues 19–32) would be approximately 21 Å, or, if all residues 16–32 of the MRP were α-helical, 29 Å. Model lipid bilayer membranes have been found to span distances of around 40 Å [20], which would be longer than the length of an MRP α-helix. If, however, the MRP could undergo a transition to an all-extended (β) structure [19], the length of this segment would increase to approximately 63 Å, allowing this segment to span the bilayer. In this case, the pores would be lined by multiple MRP domains, each extending down through the membrane from individual PNC-27-HDM-2 complexes at the outer surface of the membrane. 

The results of our experiments on the temperature dependence of PNC-27-induced tumor cell killing (Figure 6) suggest that, as with pore-forming proteins such as streptolysin, PNC-27 induces pore formation in two discrete steps: a binding step in which it complexes with HDM-2 in the cancer cell membrane and a subsequent diffusion step in which the PNC-27-HDM-2 complexes fuse together in the cell membrane to form the pores. However, the driving force for pore formation induced by streptolysin appears to be a general affinity for lipid and cholesterol in the lipid bilayer of the cell membrane [21]. In contrast, pore formation induced by PNC-27 in the cancer cell membrane is due to its affinity for membrane-bound HDM-2 and has no apparent relationship to affinity for lipid.

Implications for Cancer Therapy. In contrast to most other anti-cancer agents, PNC-27 induces tumor cell necrosis by inducing transmembrane pore formation which is independent of intracellular processes, e.g., it does not depend on the presence of specific signal transduction pathways, the action of p53 and/or of other apoptosis-inducing agents, and/or avoidance of MDR gene products but depends only on the expression of HDM-2 in the cancer cell membrane. This feature gives this peptide the ability to kill multiple types of cancers regardless of what pro-mitotic intracellular processes occur in these cells. It also differs from other anti-cancer agents whose purpose is to block the binding of p53 to HDM-2 in the nucleus allowing p53 to function in activating apoptosis in these cells [22]. These agents are effective in cancer cells that express wild-type p53 but cannot be used to treat cancer cells that contain homozygously deleted or homozygously mutated p53. In contrast, PNC-27 has been found to kill cancer cell lines that are p53 homozygously deleted [1,2]. In addition, at least some of these agents appear to inhibit the growth of normal cells [23]. We have found that PNC-27 does not block the viability or growth of many different untransformed cell lines, none of which express any appreciable levels of HDM-2 in their membranes, and has been found not to have off-target effects in vivo [16,24]. Thus, this peptide appears to be a potentially strong and effective anti-cancer agent in treating human cancers by a novel mechanism.

## Data Availability

The data presented in this study are available in article and supplementary material.

## Supplementary Materials

The following supporting information can be downloaded at: https://www.mdpi.com/article/10.3390/biomedicines10050945/s1, Figure S1: TEM of cancer and normal cells treated with control peptide PNC-29 and effective peptide PNC-27, respectively. MIAPaCa-2 cancer cells were treated with PNC-29 (A: whole cells view on the left and B: cytoplasmic view on the right). AG13145 untransformed human primary fibroblasts (C) and BMRPA1 normal pancreatic acinar cells (D) were treated with PNC-27. Intact plasma membrane and mitochondria is observed in all control TEM studies with cancer cells treated with control peptide PNC-29 or untransformed cells treated with PNC-27; Figure S2: Immuno-TEM of streptolysin O (SLO) pores. A: Human pancreatic cancer MIAPaCa-2 cells were treated with SLO, fixed and followed by staining with primary Ab against SLO, SLO mAb IgG, and a secondary Ab, 6 nm-gold-GαM IgG. B: The electron micrograph on the left shows ring structures of the SLO pore on the plasma membrane, which are magnified on the right (bars indicating magnification). Multiple gold particles decorate the rings, which indicate the location of SLO molecules.
