# Supplementary material for: PNC-27, a Chimeric p53-Penetratin Peptide Binds to HDM-2 in a p53 Peptide-like Structure, Induces Selective Membrane-Pore Formation and Leads to Cancer Cell Lysis
Source: Biomedicines. 2022 Apr 20;10(5):945. doi: 10.3390/biomedicines10050945 (PMC9138867; doi:10.3390/biomedicines10050945)
Supplement: Supplementary file 1 [file biomedicines-10-00945-s001.zip › biomedicines-1679073-supplementary.pdf]

Supplemental Figure S1.

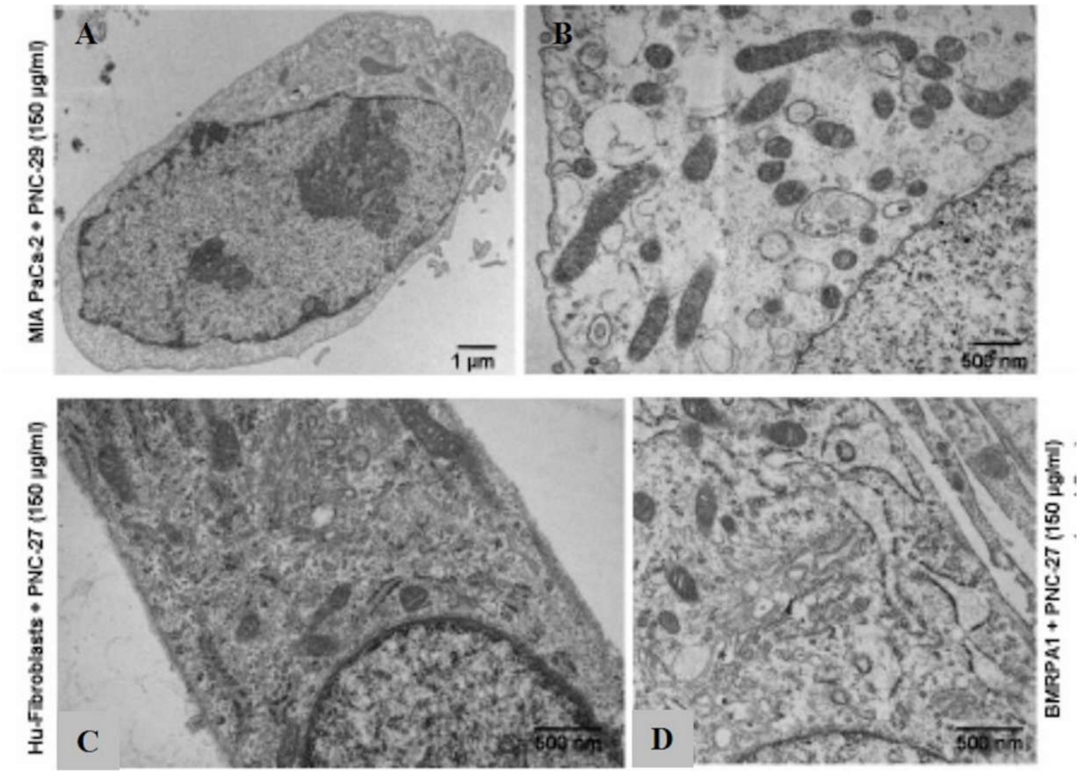

Supplemental Figure S1: TEM of cancer and normal cells treated with control peptide PNC-29 and effective peptide PNC-27, respectively. MIAPaCa-2 cancer cells were treated with PNC-29 (**A**): whole cells view on the left and (**B**): cytoplasmic view on the right). AG13145 untransformed human primary fibroblasts (**C**) and BMRPA1 normal pancreatic acinar cells (**D**) were treated with PNC-27. Intact plasma membrane and mitochondria is observed in all control TEM studies with cancer cells treated with control peptide PNC-29 or untransformed cells treated with PNC-27.

Supplemental Figure S2.

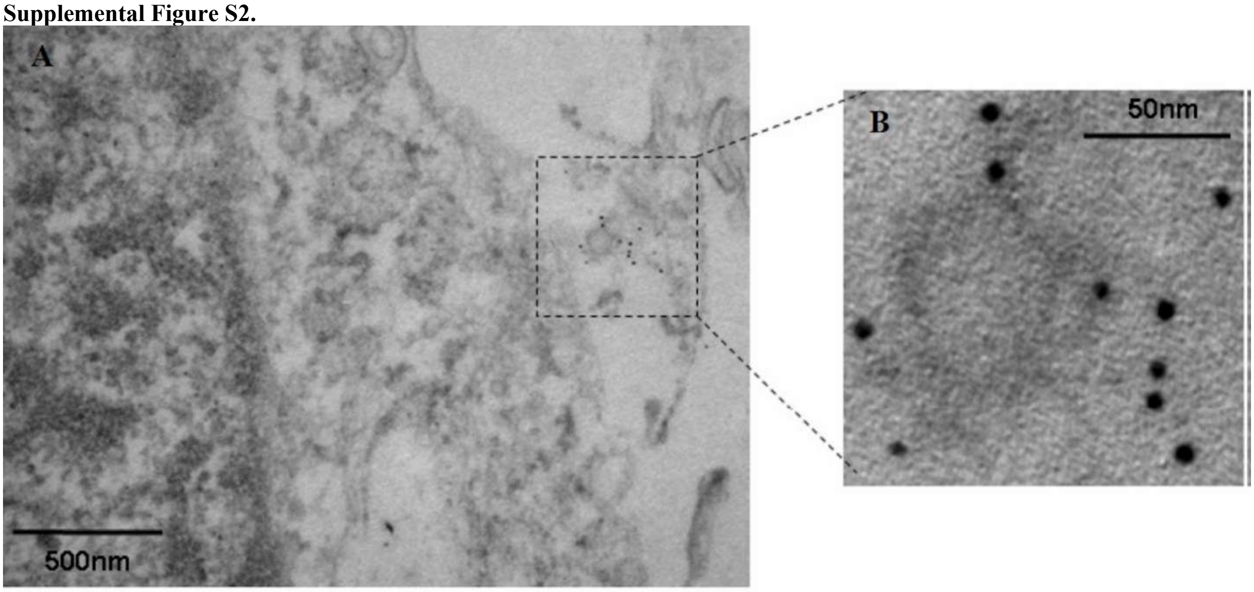

Supplemental Figure S2: Immuno-TEM of streptolysin O (SLO) pores. (**A**)**:** Human pancreatic cancer MIAPaCa-2 cells were treated with SLO, fixed and followed by staining with primary Ab against SLO, SLO mAb IgG, and a secondary Ab, 6 nm-gold-GαM IgG. (**B**)**:** The electron micrograph on the left shows ring structures of the SLO pore on the plasma membrane, which are magnified on the right (bars indicating magnification). Multiple gold particles decorate the rings, which indicate the location of SLO molecules.
